# BRILIA: Integrated Tool for High-Throughput Annotation and Lineage Tree Assembly of B-Cell Repertoires

**DOI:** 10.3389/fimmu.2016.00681

**Published:** 2017-01-17

**Authors:** Donald W. Lee, Ilja V. Khavrutskii, Anders Wallqvist, Sina Bavari, Christopher L. Cooper, Sidhartha Chaudhury

**Affiliations:** ^1^Biotechnology HPC Software Applications Institute (BHSAI), Telemedicine and Advanced Technology Research Center, U.S. Army Medical Research and Materiel Command, Fort Detrick, MD, USA; ^2^Molecular and Translational Sciences, U.S. Army Medical Research Institute of Infectious Diseases, Frederick, MD, USA

**Keywords:** B-cell receptor (BCR), repertoire, annotation, lineage, VDJ, somatic hypermutation (SHM)

## Abstract

The somatic diversity of antigen-recognizing B-cell receptors (BCRs) arises from Variable (V), Diversity (D), and Joining (J) (VDJ) recombination and somatic hypermutation (SHM) during B-cell development and affinity maturation. The VDJ junction of the BCR heavy chain forms the highly variable complementarity determining region 3 (CDR3), which plays a critical role in antigen specificity and binding affinity. Tracking the selection and mutation of the CDR3 can be useful in characterizing humoral responses to infection and vaccination. Although tens to hundreds of thousands of unique BCR genes within an expressed B-cell repertoire can now be resolved with high-throughput sequencing, tracking SHMs is still challenging because existing annotation methods are often limited by poor annotation coverage, inconsistent SHM identification across the VDJ junction, or lack of B-cell lineage data. Here, we present B-cell repertoire inductive lineage and immunosequence annotator (BRILIA), an algorithm that leverages repertoire-wide sequencing data to globally improve the VDJ annotation coverage, lineage tree assembly, and SHM identification. On benchmark tests against simulated human and mouse BCR repertoires, BRILIA correctly annotated germline and clonally expanded sequences with 94 and 70% accuracy, respectively, and it has a 90% SHM-positive prediction rate in the CDR3 of heavily mutated sequences; these are substantial improvements over existing methods. We used BRILIA to process BCR sequences obtained from splenic germinal center B cells extracted from C57BL/6 mice. BRILIA returned robust B-cell lineage trees and yielded SHM patterns that are consistent across the VDJ junction and agree with known biological mechanisms of SHM. By contrast, existing BCR annotation tools, which do not account for repertoire-wide clonal relationships, systematically underestimated both the size of clonally related B-cell clusters and yielded inconsistent SHM frequencies. We demonstrate BRILIA’s utility in B-cell repertoire studies related to VDJ gene usage, mechanisms for adenosine mutations, and SHM hot spot motifs. Furthermore, we show that the complete gene usage annotation and SHM identification across the entire CDR3 are essential for studying the B-cell affinity maturation process through immunosequencing methods.

## Introduction

B cells synthesize transmembrane proteins called B-cell receptors (BCRs) that recognize foreign antigens. The binding of BCRs with an antigen activates B cell clonal expansion and somatic hypermutation (SHM), which increases the likelihood of synthesizing high-affinity BCRs that are later secreted as antibodies into the blood [see Ref. (1) for a review on affinity maturation]. Understanding how antigen-specific antibodies are produced can aid the development of effective vaccines, for instance, by showing which BCR genes become enriched in vaccinated subjects (2–6) or by measuring the extent of affinity maturation in response to vaccination. Although thousands of BCR sequences from B cell repertoires can be obtained with high-throughput sequencing (7), tracking SHM among maturing B cells remains a challenge (8). The standard approach for processing BCR sequences is to first annotate the Variable (V), Diversity (D), and Joining (J) segments of the BCR gene, then cluster clonally related sequences, and finally construct a lineage tree for each cluster (8–11). Each step is typically carried out using separate algorithms, which can yield results that are at odds with the biological mechanisms that underlie SHM or affinity maturation. Therefore, developing algorithms that provide VDJ annotations that agree with clonal expansion and SHM behaviors is critical for accurately characterizing B-cell repertoires.

The BCR consists of a heavy chain and a light chain. During B-cell development, functional BCR genes for the heavy and light chain are formed *via* the recombination of V, D, and J gene segments within the chromosomal DNA, mediated by recombinase enzymes RAG1 and RAG2 (12, 13). The heavy chain of the BCR uses the V, D, and J segments, and the light chain uses a separate set of only V and J segments, giving rise to a baseline combinatorial diversity of BCRs. The VDJ junction of the heavy chain forms the highly variable complementarity determining region 3 (CDR3) loop structure that plays a critical role in antigen recognition (14). Further diversity is introduced in the VDJ junction at the joining regions between the gene segments through deletions of gene edges by nucleases (15), creation of palindromic sequences called palindromic nucleotides (P-nts), and insertions of non-templated nts (N-nts) by terminal deoxynucleotidyl transferase (TDT) (16–18) [see Ref. (19) for a review on VDJ recombination]. A contiguous sequence of P- and N-nts is referred to as an N region. A final level of BCR diversity is introduced through SHM that is mediated by deaminases and error-prone DNA repair enzymes, which can obscure the original VDJ genes [see Ref. (20) for a review on SHM]. The combinatorial diversity of germline gene recombination, the variation that is introduced by insertions and deletions in the VDJ junction, and the subsequent accumulation of SHMs during affinity maturation pose major challenges to BCR gene annotation.

High-throughput sequencing focused on the heavy chain CDR3 is becoming a common tool for rapidly characterizing the entire B-cell repertoire from a single sample—an inexpensive alternative to more costly single-cell sequencing approaches (21). For a given individual, the number of unique B cells is estimated to be greater than 10^7^ (22), and analysis of antigen-specific B cells may require sequencing of up to 10^4^ or 10^5^ unique B cells (23). The Illumina deep-sequencing technology is capable of providing sufficient sequencing depth to capture this repertoire in its entirety for sequence read lengths of ~150 bp (24). However, the relatively short reads, which capture the complete CDR3 sequence but exclude much of the V and J regions (including CDR1 and CDR2), present additional challenges to BCR gene annotation and SHM characterization.

Most existing annotation algorithms use a sequence alignment-based approach to resolve the V, D, and J gene segments within a given BCR sequence. The most widely used algorithms is ImMunoGeneTics (IMGT)’s VQUEST with JunctionAnalysis (VQUEST + JA) (25–28), which finds annotations for the V, J, and D genes (in this order) that maximize the sequence alignment scores with respect to a database of unmutated, or germline, sequences. VQUEST + JA also use the conserved 104Cys and 118Trp/Phe residues surrounding the CDR3 [which are residues numbered according to IMGT’s unique numbering system (29)] to fine-tune the annotations (28). Examples of other algorithms that use similar alignment-based annotation methods include IgBlast (30), SoDA (31), JointML (32), JOINSOLVER (33), VDJSeq-Solver (34), MiXCR (35), IMSEQ (36), and IgSQUEAL (37). A different strategy, hidden Markov modeling (HMM), uses statistical models and probability matrices to calculate the most likely series of events leading to each sequence. Algorithms that use HMM are SoDA2 (38), iHHMune-align (39), JointHMM (32), and *partis* (40). Most BCR annotation algorithms provide high accuracy in annotating V and J segments, but struggle to annotate the N and D segments that define the critical CDR3. The D genes are especially difficult to annotate because they are short in length (e.g., 9–18 nt for mice) and can be inverted (32, 41), severely truncated, blended with the N regions, and highly mutated. Thus, an effective method for annotating the D gene is to first find the least mutated or most ancestral BCR sequence among the clonally related BCR sequences. However, employing this strategy requires that B-cell lineages be determined concurrently with VDJ annotations.

B-cell lineages are typically determined separately from BCR annotation, despite their common biological basis. A common way to identify clonally related sequences is to cluster sequences with the same V(D)J annotation and CDR3 length and with a high level of BCR nt sequence similarity based on a Hamming distance cutoff (11, 27, 36, 42). Although annotation-free clustering has been used (43), determining lineage trees per cluster is difficult without the full VDJ gene annotations. After clustering, lineage trees are assembled per cluster by using algorithms such as MEGA5 (44), PHLYPIS (45), ImmuniTree (46), or IgTree (47). The assumptions underlying the annotation, clustering, and tree assembly algorithms may be mutually inconsistent, leading to issues such as inadvertent segmentation of long lineage trees owing to divergent VDJ annotations or assembly of binary trees that do not properly reflect B-cell clonal expansion. B-cell lineage trees can be highly branched because a group of identical B cells can give rise to multiple lineages when undergoing SHM. Integrated software applications, such as Change-O (10) and RevertToGermline + AnnotateTree (48), streamline the process of assembling lineage trees that are consistent with the annotations, but in these methods, the lineage tree information is not used to improve annotations.

Evaluating the performance of BCR annotation tools is challenging because the true VDJ annotations are not known in real-life BCR sequencing data. As a result, most annotation tools are benchmarked against simulated BCR repertoires created either through in-house simulations or tools such as IgSimulator (49). However, when processing our own BCR data sets, existing annotation algorithms had difficulty yielding consistent nt substitution frequencies across all V, D, and J segments. There is no biological basis for this inconsistency—SHM-inducing enzymes that are responsible for the substitution patterns, such as activation-induced cytidine deaminase (commonly referred to as AID) (50–53), do not necessarily discriminate between V, D, and J segments. Therefore, we used the correlation between the SHM nt substitution patterns of the V segment and those of the DJ segments as a proxy for the overall annotation quality of real-life BCR repertoires.

In this study, we present B-cell repertoire inductive lineage and immunosequence annotator (BRILIA), a BCR annotation algorithm that concurrently annotates genes, clusters sequences, and assembles lineage trees. BRILIA refines annotations by exploiting mechanistic biases in SHM patterns, N region nt compositions, and directionality of N region synthesis by TDT on the coding versus non-coding DNA strand. We benchmarked BRILIA by processing short 125-bp sequences from simulated human and mouse BCR repertoires and real-life repertoire data obtained from splenic germinal center B cells isolated from C57BL/6 mice. BRILIA identified more highly branched lineage trees and obtained more consistent SHM patterns across the VDJ segments when compared to currently available methods. We demonstrate how BRILIA annotations and lineage trees can be applied in research on affinity maturation, VDJ gene usage frequencies, and SHM mechanisms and hot spot motifs.

## Materials and Methods

### Obtaining the Database for VDJ Germline Genes

Human and mouse VDJ germline genes were downloaded from the international IMGT database (http://www.imgt.org) (54–61). When annotating C57BL/6 mouse data sets, only genes obtained from the same mouse strain were kept to prevent strain bias of VDJ gene alleles (41). Pseudogenes, which are germline genes with stop codons or frame shift mutations, were included in the database since their role in producing functional VDJ is still debated (62, 63). However, only annotations without any stop codons and frame shift errors, referred to as productive VDJ junctions, were analyzed at the end. We included inverted D genes by default because they have been observed occasionally (32, 33, 64, 65). Users are given the option to disallow inverted D matching because it may not significantly improve the annotation results (66). We used the IMGT gene nomenclature, but added an “r” before the family name (e.g., rIGHD01-1*01) for the inverted D genes.

### Simulating BCR Repertoires for Benchmarking Annotation Algorithms

To benchmark our annotation method, we simulated a BCR repertoire so that the true annotations are known. The purpose of this simulated repertoire is to gauge the ability of an algorithm to identify the actual VDJ genes and SHM events and not necessarily to simulate the actual usage frequencies of VDJ genes. Because different genes are used with lesser or higher frequencies than others in real-life repertoires (32, 33), replicating this feature in simulated repertoires would have limited the VDJ combinations that were tested. The simulated sequences preserved other details such as the frequent C ➔ T and G ➔ A mutations mediated by AID (50–53), preferential occurrence of A mutations over T mutations (referred to as strand-bias mutations) (67, 68), and biased N region nt compositions (69, 70). We refer to the N region between the V and D segments as N_VD_ and that between D and J segments as N_DJ_. Only productive VDJ junctions were generated to ensure a fair comparison of each algorithm’s core functions. A total of 1,000 unmutated, or “germline,” sequences were generated. To simulate SHM, five descendant sequences were generated for each germline sequence by mutating 5 nt at non-repeating locations, five times. The combined germline and mutated sequences (a total of 6,000 sequences) were labeled as “clonally expanded” sequences. More details about the BCR simulations are provided in the Supplementary Material. Simulated human and mouse BCR sequences can be found in Datasheets S1 and S2 in Supplementary Material, respectively.

### Extracting BCR Sequences from C57BL/6 Mice Spleens

Germinal center B cells were isolated from wild-type C57BL/6 mice purchased from Jackson Labs. In brief, single-cell suspensions from homogenized spleens were washed using FACS buffer (phosphate-buffered saline, 0.5% bovine serum albumin, and 2 mM ethylenediaminetetraacetic acid; Corning, Sigma), lysed with red blood cell buffer (Sigma), and then counterstained with the following B-cell antibodies: B220, IgM, IgD, IgG1, CD38, CD138, and GL-7 (BD Biosciences). All samples were Fc-blocked (anti-CD16/CD32) and stained to evaluate viability (live/dead aqua, Invitrogen) prior to antibody staining. Highly purified (> 90%) Spt-GC B cells (B220^+^GL7^+^CD95^+^CD38low) were then isolated using cell sorting on a BD Aria II. Subsequently, purified Spt-GC B cells were pelleted by centrifugation, snap frozen, and then shipped to Adaptive Biotechnologies for DNA extraction and next-generation sequencing of the murine VDJ loci. BCR sequence information was obtained from amplicons beginning within FR3 of the V gene and ending just 3′ of the complete VDJ junction. Each sequence was trimmed to 125 bp maintaining the last 3 nt of the sequence codes for the conserved 118Trp (TGG) of the CDR3. For each unique sequence, template counts were also provided, which reflect the number of B cells that had a copy of a particular BCR gene (71). Animal work was conducted under a United States Army Medical Research Institute of Infectious Diseases (USAMRIID) Institutional Animal Care and Use Committee-approved protocol in compliance with the US Animal Welfare Act, Public Health Service Policy, and other federal statutes and regulations relating to animals and experiments involving animals. The facility in which this research was conducted (USAMRIID) is accredited by the Association for Assessment and Accreditation of Laboratory Animal Care, International and adheres to principles stated in the Guide for the Care and Use of Laboratory Animals, National Research Council, 2011. Real-life BCR sequences used in this work can be found in Datasheet S3 in Supplementary Material.

## Stepwise Procedures

### BRILIA Algorithm Overview

**Figure 1:**
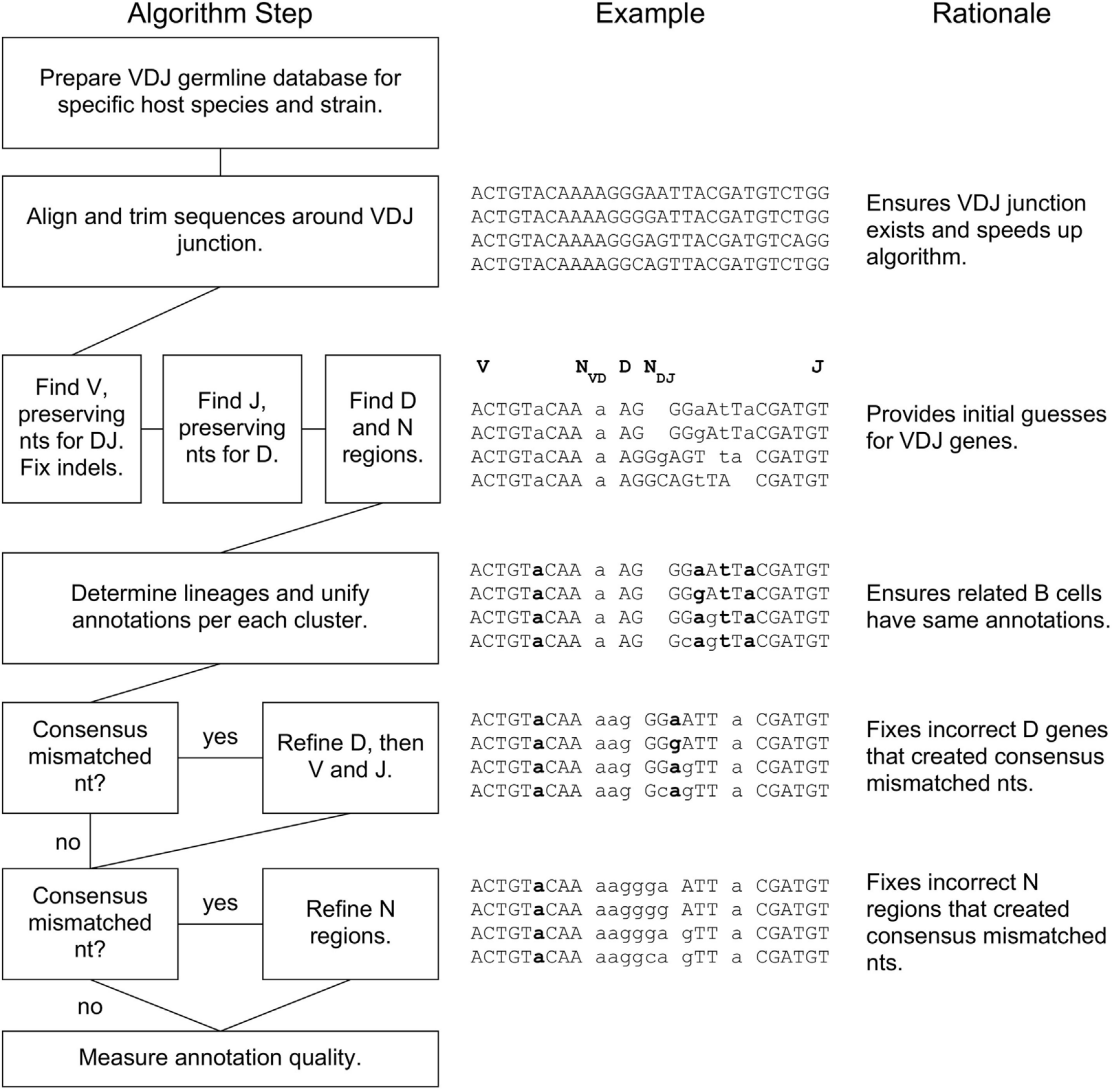
**BRILIA flowchart with examples and rationales for each step**. For the sample sequences in the middle, the V, N_VD_, D, N_DJ_, and J segments are separated by a space, where a double space indicates a lack of N region (e.g., N_DJ_ is absent initially). A lowercase letter is either an N nucleotide (nt) or a mismatched nt with respect to the germline genes, and a bolded letter is a consensus mismatched nt.

A flowchart of our BRILIA annotation algorithm is shown in Figure 1, along with an example of the process and rationale behind each major step. The key features of our annotation strategy are the alignment strategy that accommodates variable SHM rates per sequence, preservation of nts during alignment that prevent “no D” results, lineage-based clustering, unification of annotations within a cluster, D inverse searches, and refinement steps for D and N regions. BRILIA is written in MATLAB (MathWorks), and all source codes and input files used in this study are available on request.

### Defining the Alignment Scoring Method

The initial VDJ annotations rely on aligning sequences to the germline sequences and maximizing the total alignment score for the VDJ segments. We used a custom alignment scoring method defined as

(1) $$\text{Score}=\sum_{i}C_{i}^{2}-\sum_{j}M_{j}^{2},$$

where C*_i_* and M*_j_* are the numbers of consecutively matched and mismatched nts, respectively, in a segment. For example, if the result of a sequence alignment is “AGtTTcC,” where lowercase letters represent mismatched nts, the alignment score would be computed as 2^2^ − 1^2^ + 2^2^ − 1^2^ + 1^2^ = 7. To prevent SHM from severely affecting the alignment score, we add a mismatch leniency rule so that point mutations (but not consecutively mismatched nts) can elongate consecutively matched segments. By using the above example and allowing for one mismatch, the score would now be computed as (2 + 0 + 2)^2^ − 1^2^ + 1^2^ = 16. Note that despite having another point mutation near the 3′ end, we elongate segments near the 5′ end first because SHM occurs more frequently near the 5′ end (72).

For the V segment, we set the mismatch leniency to be as high as 15% of the V segment length, although the actually mutation% is often less. For the D and J segments, we set a mismatch leniency rule to have the same mutation% as what was found for the V segment, although the D and J segments in the CDR3 can accumulate more mutations (73). Since the first objective of BRILIA is to identify the least mutated sequence, setting a higher mutation% for the D and J segments is not necessary.

### Step 1: Matching VDJ Genes and Correcting V Gene Indel Errors

For a given VDJ sequence, we matched the V gene first, but with the condition that the last 9 nt were preserved for matching the D and J genes. For instance, given 125 nt in a sequence, only the first 116 nt would be used to determine a V gene. This nt preservation step prevents ‘overmatching’ a V gene such that the J and D genes cannot be resolved. We also corrected for V gene insertions/deletions (indels) that occurred before the 104Cys, because indels here are likely caused by sequencing errors (39). We did not correct for indels in the CDR3 because indels can be caused by real VDJ recombination events.

Once a V gene match was found, we preserved 3 nt to the right of the V gene segment and then determined the J gene with the remaining nts. For instance, if the first 100 of 125 nt were matched to a V gene, then nts 101 to 103 were preserved, while the last 22 nt were used to match the J gene. After determining a J gene, all remaining nts were used to match the D gene. Any nts not assigned to a V, D, or J gene were treated as P-nts) and/or non-templated (N) nts, which were then assigned to their respective N_VD_ or N_DJ_ region.

### Step 2: Assembling Lineage Trees, Clustering Sequences, and Unifying VDJ Annotations

We next clustered the sequences by using lineage trees. Sequences with the same CDR3 lengths and VJ gene family numbers were clustered before constructing lineage trees because the latter process is more computationally expensive. Sequences were considered related to each other if they were within a certain sequence similarity distance. We used a custom distance metric, referred to here as the SHM distance, which resembles the Hamming distance but includes the following adjustments:

(1) Consecutively mismatches M number of nts add M^2^ to the SHM distance instead of merely M. This increases the distance between clonally unrelated sequences that may have similar VDJ genes but slightly different N regions.
(2) Frequently observed [C ➔ T, G ➔ A, A ➔ G, A ➔ T] mutations reduce the SHM distance by 0.5 units per mutation; less frequently observed [T ➔ C, A ➔ C] mutations have no effect; and all other rarer mutations increase the SHM distance by 0.5 units. These adjustments create asymmetry in distances between two sequences, which helps with determining parent–child relationships.

Example: If Seq1 = ACGCTT and Seq2 = AttgTT, then the SHM distance is 3^2^ + [−0.5 + 0.5 + 0.5] = 9.5, assuming Seq1 is the parent. If Seq2 is assumed to be the parent, the SHM distance is 3^2^ + [0 + 0.5 + 0.5] = 10. In this case, we would assume Seq1 is the parent of Seq2.

Parent–child sequence relationships were determined within each cluster by using a nearest-distance method. The initial linkages generate cyclic dependencies (e.g., Seq1 ➔ Seq2 ➔ Seq1) because the root has not yet been assigned. For each independent tree cluster, the root sequence was determined as that which is involved in the cyclic dependency and has the smallest total SHM distance to all other sequences in that cluster. Any ties in the root sequence determination were broken by assigning the sequence with the highest VDJ alignment scores as the root. In an iterative process, the root of each small cluster was linked to any sequence in another cluster, as long as it did not exceed the SHM distance cutoff equal to 3% of the sequence length. Note that this cutoff distance can be adjusted by the user.

Finally, we defined a BRILIA cluster as a group of sequences that shared a common root sequence. The VDJ annotations and N region demarcations for each cluster were unified to match those of the root sequence. The lineage tree was rerooted only if another sequence served as a better root sequence based on a closer distance to the predicted germline genes. Hereafter, any annotation refinements to a cluster were applied to all sequences in the cluster.

### Step 3: Refining D Annotations within a Cluster

After unifying the VDJ annotations per cluster in the prior step, annotation errors can become more apparent. A common indication of annotation error is when all sequences have a mismatched nt in the CDR3 that does not match with the germline sequence, which we will refer to as a consensus mismatched nt (see Figure 1, bold letters in example sequences). If a consensus mismatched nt was present in the framework region of the V gene (Vframe), then we assumed that the consensus mismatched nts in the CDR3 were a byproduct of real SHMs. Otherwise, we assumed that the consensus mismatched nts occurred by a suboptimal annotation and attempted to remove them by refining the D gene alignment. Since changing the D gene results also changes the N regions, we had to determine whether the resulting N region compositions agreed with the actions of TDT, which prefers to add A and G (69, 70, 74). We computed the probability that an N region is created by TDT relative to purely random nt insertion, denoted as *P_TDT_*, using the equation below.

(2) $$P_{\text{TDT}}=\frac{\prod_{j=1}^{L}P_{X(j)}}{0.25^{L}+\prod_{j=1}^{L}P_{X(j)}},$$

where L is the length of the N region and *P_*X(j)*_* is the probability that TDT adds nt *X* (A, C, G, T) at position *j* in the N region. We assumed that nts that are not associated with TDT activity has an occurrence probability of 0.25, whereas nts added by TDT have the following occurrence probabilities: *P_A_* = 0.25, *P_C_* = 0.08, *P_G_* = 0.60, and *P_T_* = 0.07 (see Figure S2 in Supplementary Material). These probability values were obtained from the N regions of our data set from mice, after converting these regions to their complement sequences if there were more CT content than AG content, which should better capture the TDT-mediated DNA elongation patterns (69) (see Supplementary Material). We note that the *P_X_* values are reported for healthy mice, and we do not expect these to vary much across subjects unless there are abnormal conditions [e.g., nt pool imbalance (75)].

We next calculated a custom N region likelihood score, or *Nscore*, by using the following equation:

(3) $$\text{Nscore}={\left[P_{\text{TDT}}L\right]}^{2}$$

The *Nscore* was calculated for both the normal and complement sequences of each N region, and only the higher score was retained. A different D gene annotation was accepted only if it increased the sum of the VDJ alignment scores and the Nscores for N_VD_ and N_DJ_. If a consensus mismatch persisted, then we evaluated whether this was caused by the incorrect demarcation of the N regions, as discussed next.

### Step 4: Refining N Regions within a Cluster

Improper demarcation of N regions can also cause consecutively mismatched nts to exist in the CDR3 (marked as bold lower case letters in a sequence alignment), which can be fixed by redefining where the VDJ gene segments are. For any three consecutively mismatched nts near gene segment edges, we automatically reassigned the edges to the N regions because such events are likely caused by annotation errors. For example, if a V gene ended with “5′-TG**agg**GG,” then “agggg” was automatically added to the N_VD_ region. For all other cases, we checked whether the gene segment edges had compositions that reflected TDT-mediated nt insertion. Several examples cases are provided below.

- If no N region is initially present and trimming would create one, then we checked whether *P_TDT_* of the trimmed nts was >0.50. For instance, if a V gene ended with “5′-TGCA**g**GG,” then *P_TDT_* for “**g**GG” is 0.93 and therefore, “ggg” became the N_VD_ region. If a V gene ended with “5′-CA**t**ATC,” then *P_TDT_* for “**t**ATC” is 0.40, and therefore, no trimming was performed.
- If an N region is initially present, then we calculate whether adding the edge nts to the N region would increase *P_TDT_*. For instance, if the V gene ended with “5′-TGCA**g**GG” and the N_DV_ region was “ccc,” adding “**g**GG” to “ccc” would have created an unfavorable “gggccc” in the N_VD_ region with a reduction in P_TDT_ from 0.93 to 0.31; hence, no trimming was performed.

## Results

### VDJ Annotation of Simulated BCR Repertoire Data

Obtaining accurate gene annotations is essential to measuring gene usage frequency (76), tracking affinity maturation and selection pressure (77–79), and studying SHM-associated enzyme activities (51–53, 80–83). Because the true accuracy of VDJ annotation cannot be determined when using real-life BCR repertoires, we created a synthetic repertoire for benchmarking purposes. We compared our annotations with those of two recently updated algorithms, VQUEST + JA (25–28) and *partis* (40). If an algorithm suggested multiple VDJ annotations, we retained only the first suggestion.

We compared how well the algorithms could obtain an exact match to the actual gene (up to the gene allele number) and also a degenerate match to any gene name that contains ≥98% of the same nts (Tables 1 and 2). An example of a degenerate match is when “ATTAACTA” of IGHD1-1*01 was used generate a BCR sequence and the annotation suggested IGHD1-1*02, which has the same nts. Tables 1 and 2 compare the gene matching performances of the three algorithms on human and mouse sequences, respectively, for both germline and clonally expanded sequences. However, we did not annotate the mouse repertoire with *partis* because it was intended for annotating human BCR genes only (40).

**Table 1:** **Annotation accuracy in simulated human B-cell receptor (BCR) repertoire**.

		B-cell repertoire inductive lineage and immunosequence annotator (BRILIA)		VQUEST + JA		*Partis*	
**Germline** 1,000 sequences	V exact match	826	83%	393	39%	481	48%
	D exact match	830	83%	613	61%	660	66%
	J exact match	837	84%	530	53%	686	69%
	VDJ exact match	562	56%	139	14%	231	23%
	V degen match	1,000	100%	862	86%	969	97%
	D degen match	956	96%	808	81%	890	89%
	J degen match	985	99%	843	84%	938	94%
	VDJ degen match	941	94%	655	66%	857	86%
**Clonally expanded** 6,000 sequences	V exact match	4,045	67%	1,928	32%	2,442	41%
	D exact match	4,359	73%	2,990	50%	3,419	57%
	J exact match	4,548	76%	2,783	46%	3,777	63%
	VDJ exact match	2,291	38%	508	8%	936	16%
	V degen match	5,159	86%	4,570	76%	5,272	88%
	D degen match	4,979	83%	3,893	65%	4,575	76%
	J degen match	5,477	91%	4,546	76%	5,195	87%
	VDJ degen match	4,059	68%	2,670	45%	3,827	64%

**Table 2:** **Annotation accuracy in simulated mouse (C57BL/6) B-cell receptor (BCR) repertoire**.

		B-cell repertoire inductive lineage and immunosequence annotator (BRILIA)		VQUEST + JA	
**Germline** 1,000 sequences	V exact match	970	97%	808	81%
	D exact match	796	80%	382	38%
	J exact match	965	97%	558	56%
	VDJ exact match	751	75%	184	18%
	V degen match	998	100%	942	94%
	D degen match	947	95%	833	83%
	J degen match	993	99%	918	92%
	VDJ degen match	938	94%	753	75%
**Clonally expanded** 6,000 sequences	V exact match	5,271	88%	4,425	74%
	D exact match	4,088	68%	1,721	29%
	J exact match	5,604	93%	3,244	54%
	VDJ exact match	3,428	57%	7,65	13%
	V degen match	5,474	91%	5,214	87%
	D degen match	4,910	82%	3,878	65%
	J degen match	5,777	96%	5,275	88%
	VDJ degen match	4,351	73%	3,129	52%

The annotation accuracy of the germline sequences reflects how well each algorithm works in the best-case scenario where SHM does not obscure VDJ genes. This accuracy is important because if a germline sequence exists within a cluster of clonally related sequences, then the corresponding annotation will be applied to all members in the cluster. BRILIA provided the highest accuracy of matching, followed closely by *partis* and VQUEST + JA (Tables 1 and 2, “Germline Sequence” rows).

The annotation accuracy of clonally expanded sequences reflects how well each algorithm can annotate sequences that have undergone extensive SHM. For the simulated human BCR sequences, BRILIA achieved 83% D gene degenerate matching accuracy, compared to 65% by VQUEST + JA and 76% by *partis* (Table 1, “Clonally Expanded” rows). For the simulated mouse BCR sequences, BRILIA achieved 82% degenerate D gene matching accuracy, compared to 65% by VQUEST + JA (Table 2, “Clonally Expanded” rows). The degenerate V and J gene annotation accuracies are comparable across all algorithms, as might be expected given the relatively long lengths of the V gene segments and the limited number of J germline genes. It is important to note that the overall V and J annotation accuracies presented here are lower than those obtained in previously published other benchmark tests (37, 40); this is because previous benchmarks used the full VDJ segments (~400 bp), while we used much shorter sequences (125 bp) typical of CDR3-focused next-generation sequencing (71).

### SHM Identification Accuracy of Simulated BCR Repertoire Data

In addition to correctly annotating the VDJ genes, it is important to accurately identify SHMs within the CDR3. For each algorithm, and for sequences grouped by the same number of simulated SHMs, we first computed the accuracy of determining mutated and unmutated nts in the CDR3 (Table 3). For all algorithms, accuracy decreased as sequences accumulated more SHMs, as expected, but BRILIA retained the highest accuracy, followed by *partis* and closely by VQUEST + JA. We also computed the positive prediction rate of mutations, which reflects how many of the predicted SHMs were true. BRILIA retained the highest positive prediction rates, followed by VQUEST + JA and closely by *partis*.

**Table 3:** **Accuracy (ACC) and positive prediction rate (PPR) of identifying SHMs in the complementarity determining region 3 (CDR3) from the simulated clonally expanded BCRs**.

	Lineage generation	# of SHMs in 125-bp BCR sequence	TP	TN	FP	FN	ACC	PPR
**BRILIA human**	Germline	0	0	51,544	1,031	0	0.98	0.00
	1st	5	1,230	50,072	629	644	0.98	0.66
	2nd	10	2,433	48,146	676	1,320	0.96	0.78
	3rd	15	3,373	46,141	755	2,306	0.94	0.82
	4th	20	4,376	44,310	633	3,256	0.93	0.87
	5th	25	5,588	42,370	612	4,005	0.91	0.90
**VQUEST + JA human**	Germline	0	0	46,724	502	0	0.99	0.00
	1st	5	913	44,296	812	758	0.97	0.53
	2nd	10	1,636	41,973	844	1,645	0.95	0.66
	3rd	15	2,146	39,634	919	2,745	0.92	0.70
	4th	20	2,365	36,937	1,010	4,007	0.89	0.70
	5th	25	2,324	33,983	1,114	5,410	0.85	0.68
***partis* human**	Germline	0	0	50,685	654	0	0.99	0.00
	1st	5	1,160	48,182	1,149	665	0.96	0.50
	2nd	10	2,325	45,946	1,695	1,334	0.94	0.58
	3rd	15	3,505	43,251	2,228	2,001	0.92	0.61
	4th	20	4,655	40,405	2,828	2,686	0.89	0.62
	5th	25	5,648	37,713	3,154	3,441	0.87	0.64
**BRILIA mouse**	Germline	0	0	42,144	804	0	0.98	0.00
	1st	5	976	41,062	410	500	0.98	0.70
	2nd	10	1,976	39,518	443	1,011	0.97	0.82
	3rd	15	2,746	37,891	527	1,784	0.95	0.84
	4th	20	3,531	36,427	421	2,569	0.93	0.89
	5th	25	4,579	34,951	374	3,044	0.92	0.92
**VQUEST + JA mouse**	Germline	0	0	38,602	209	0	0.99	0.00
	1st	5	743	36,843	459	592	0.97	0.62
	2nd	10	1,407	35,471	522	1,315	0.95	0.73
	3rd	15	1,864	33,978	639	2,270	0.92	0.74
	4th	20	2,176	32,294	680	3,328	0.90	0.76
	5th	25	2,298	30,090	739	4,346	0.86	0.76

*Accuracy is computed as (TP + TN)/(TP + TN + FN + FP), where T = true, P = positive, F = false, and N = negative. PPR is computed as TP/(TP + FP). The nucleotides in the CDR3 are pooled together based on the sequence lineage (e.g., 1st descendants of the Germline B cells), prior to determining the SHM accuracy statistics. The total nucleotide count differs among algorithms because the annotation coverage differs*.

We next measured how well each BCR annotation method can identify the frequency in which one nt (X_0_) mutates to another nt (X_1_), which we will refer as the SHM propensity. The SHM propensities are not uniform, and certain X_0_ ➔ X_1_ mutations occur more frequently than others. For instance, the C ➔ T mutations (and the complement G ➔ A mutations) occur frequently because they are triggered by activation-induced cytidine deaminase (AID), which initiates the C ➔ U ➔ T substitutions (50–53). Although deaminases are known to act on specific nt sequence motifs, called hot spots, there is no evidence that they can discriminate between the V, D, and J segments of the BCR. Therefore, we expect high-quality SHM identification to show SHM propensities that are (1) consistent across the entire VDJ junction and (2) agree with deaminase-mediated nt substitution patterns.

**Figure 2:**
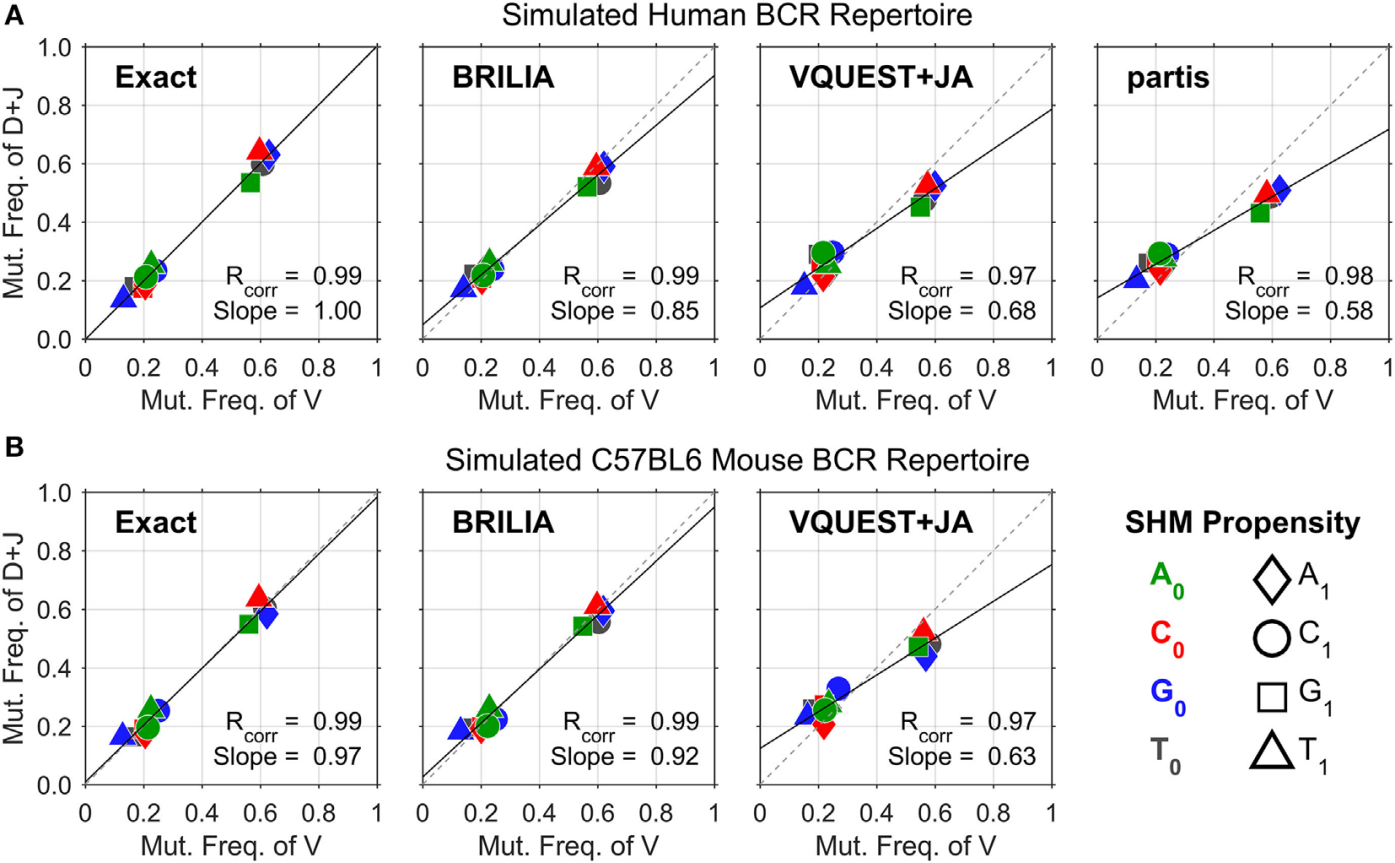
**Somatic hypermutation (SHM) propensity correlations between the V and DJ segments for simulated (A) human and (B) mouse B-cell receptor sequences**. The combination of color and shape of a data point represents a SHM propensity or the mutation frequency of nucleotide (nt) X_0_ to nt X_1_. The *x*- and *y* axes show the normalized mutation frequencies (e.g., *P_A➔T_* + *P_A➔C_* + *P_A➔G_* = 1) for the V and DJ segments, respectively. *R_corr_* is the Pearson correlation coefficient, while *Slope* is the slope of the linear regression line.

Figure 2 shows the correlation between SHM propensities for the V and DJ segments, as predicted by each method for the simulated human and mouse BCRs. All methods yielded SHM rates that are highly correlated across the V and DJ segments, as shown by the Pearson correlation coefficient (*R_corr_*) being close to 1. However, VQUEST + JA and *partis* tended to underpredict well-known SHM propensities (i.e., C ➔ T, G ➔ A, and A ➔ G) in the DJ segments, as shown by the reduced linear regression line slope (*Slope*).

Assessing annotation quality for real-life BCR repertoires is difficult because the true gene annotations are unknown. The correlation of SHM propensities across the V and DJ segments provides an alternate measure of SHM identification accuracy that does not rely on knowing the true VDJ gene assignments. One could assess the quality of VDJ gene annotations indirectly by testing if SHM propensities are consistent across the VDJ junction. Given the common biological basis for SHM across the VDJ junction, high-quality annotations should yield measures of *R_corr_* and *Slope* that approach the value of 1. However, the proper generation of these correlation metrics in real BCR repertoire data requires the determination of B-cell lineages because SHM propensities should be identified with respect to a parent–child relationship between a pair of sequences, not against a predicted germline sequence, where inherited mutations from a previous generation would be treated as new independent mutation events. For our simulated sequences, determining lineages was not as critical because mutations did not occur more than once in the same place; in other words, the SHM propensities computed from germline–child sequences would be similar to those from parent–child sequences. In real-life BCR sequences where multiple mutations can occur in the same position, the SHM propensities computed from parent–child versus germline–child sequence pairs will differ.

### SHM Identification Accuracy of Real-Life Mice BCR Repertoire Data

To test how well BRILIA performs on real-life data sets, we sequenced and analyzed 12,300 unique BCR gene sequences collected from the spleen germinal centers of C57BL/6 mice. It is important to note that these mice were not immunized, and thus, the B cells isolated from the spleen likely developed within spontaneously-formed germinal centers (Spt-GCs). Although the exact cause of Spt-GC formation is unclear, it is thought to arise for a range of reasons, from autoimmunity (84, 85) to bacterial infection or escape (86). Previous studies have suggested that Spt-GCs resemble immunization-induced GCs and spontaneous GC B cells undergo some degree of affinity maturation, including accumulation of SHMs and class switching (84).

We compared BRILIA with a method of processing BCR sequence data that entails grouping sequences with the same VDJ annotations and CDR3 lengths returned by VQUEST + JA, followed by an additional clustering step based on a sequence similarity cutoff distance (hereafter the “Standard” method). For the standard method, we used the same lineage-tree based clustering step as that used by BRILIA; the main differences between the standard and BRILIA methods lie in the alignment algorithms and annotation-based clustering step that occurs prior to the lineage-based clustering step.

**Figure 3:**
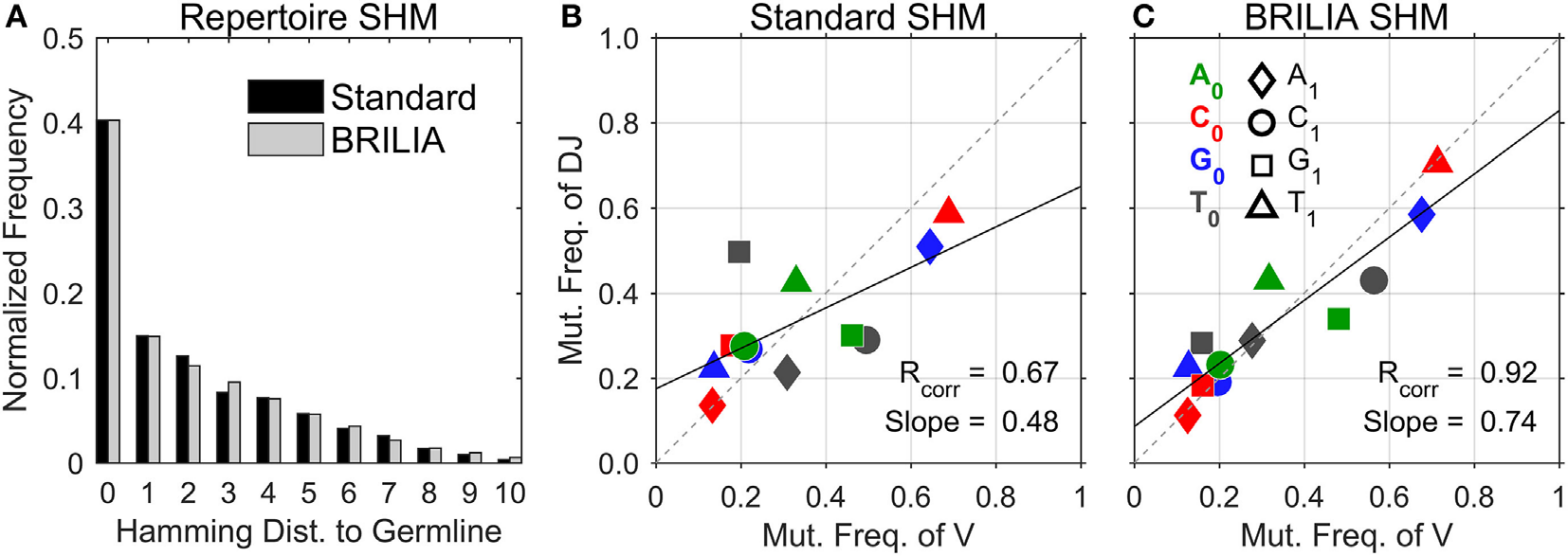
**Comparison of somatic hypermutation (SHM) identification in real-life C57BL/6 B-cell receptor repertoires between the standard method and BRILIA**. **(A)** Frequency distribution of SHMs per sequence predicted for all sequences in relation to their corresponding cluster’s germline sequence. **(B)** SHM propensity correlation returned by the standard method. Note that SHMs were determined for parent–child sequence pairs and not germline–child sequence pairs. **(C)** SHM propensity correlation returned by BRILIA.

We first describe the traditional approach of showing the SHM level of repertoires, i.e., to count the number of mutated nts in a sequence with respect to the germline sequence (Figure 3A). Both the standard method and BRILIA appear to return similar SHM frequencies. However, the correlation of SHM propensities between the V and DJ segments differ significantly between the two methods (Figures 3B,C). The standard method tended to underpredict SHMs in the DJ segments, and the T ➔ X mutation frequencies of the V segment show no correlation with those of the DJ segments (Figure 3B). In contrast, the same correlation plot based on BRILIA annotations show a high correlation between the V and DJ segments (Figure 3C). These results suggest that while BRILIA and the standard method estimate a similar *number* of SHMs, BRILIA is more accurate in identifying the SHM positions themselves.

### VDJ Gene Usages for Real-Life Mice BCR Repertoire Data

**Figure 4:**
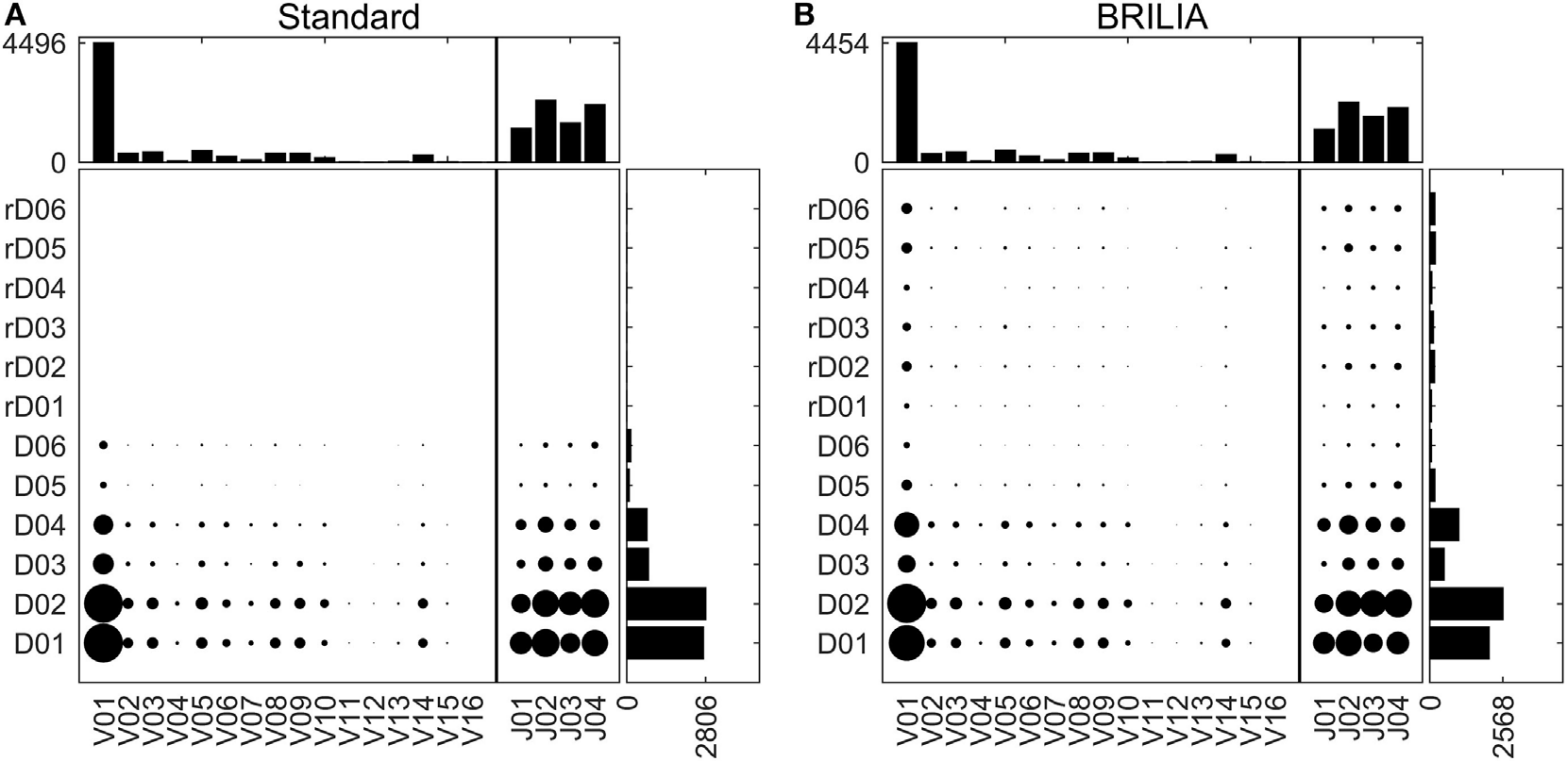
**V, D, and J gene usage frequencies**. Frequency distributions of individual VDJ gene families, and VD and DJ pairs as determined by **(A)** the standard method and **(B)** BRILIA.

Tracking VDJ gene usage is relevant for combinatory gene usage frequency studies (33, 76, 87). Figure 4 shows the VDJ gene usage frequencies in terms of both overall gene family usage frequency (top and right bar charts) and frequency of VD and DJ pairs (scatter plots). Although the standard and BRILIA methods predicted similar usage frequencies for V and J gene families (Figures 4A,B, top bar charts), their predictions for the D gene families differed substantially. BRILIA results show that IGHD4 is used twice as much as IGHD3 and that IGHD2 is used 20% more than IGHD1. In contrast, the standard method results show that IGHD1 and IGHD2 occur at similar frequencies, whereas the same applies to IGHD3 and IGHD4. Differences in D gene usages appear to arise from differences in clustering (see next section). BRILIA also returned a small number of inverted D gene annotations, although these occur less frequently than normal D gene annotations. Manual inspection of genes with inverted D annotations revealed that most were inherently ambiguous sequences, and disallowing inverted D annotations did not improve the alignment scores or correlation metrics (data not shown).

### Clustering Results for Real-Life Mice BCR Repertoire Data

**Figure 5:**
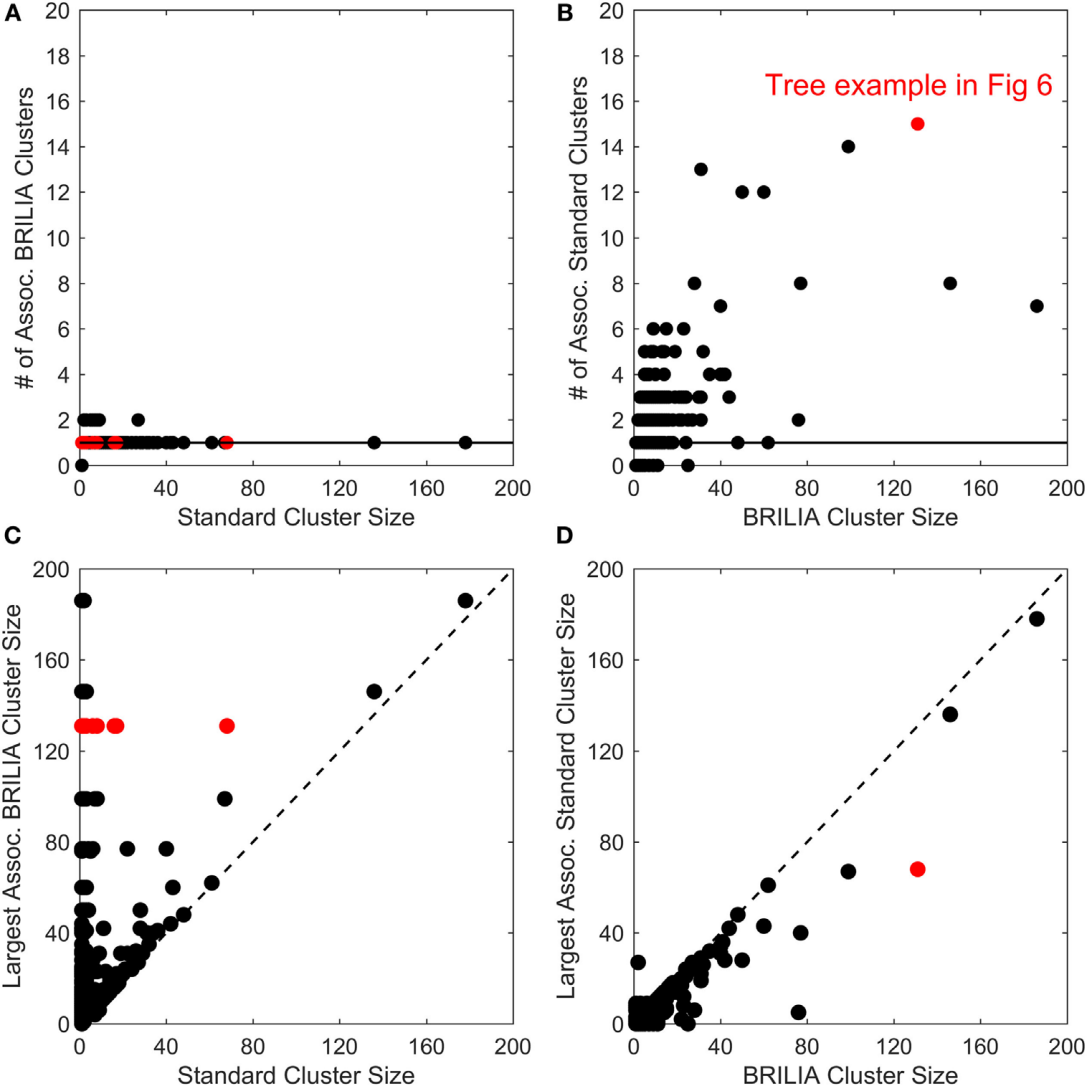
**Comparison of cluster counts and sizes between annotations made using the standard method and BRILIA**. **(A)** Number of BRILIA clusters that are associated (Assoc.) with each standard cluster, where associated clusters share at least one B-cell receptor sequence. The red dots represent clusters whose corresponding lineage trees are shown in Figure 6. **(B)** Number of standard clusters that are associated with each BRILIA cluster. **(C)** Largest BRILIA cluster size associated with each standard cluster. The dotted diagonal line (*y* = *x*) highlights differences in the associated cluster sizes between the two methods. **(D)** Largest standard cluster size associated with each BRILIA cluster.

B-cell lineage clustering can be used to describe the breadth and extent of affinity maturation and identify promising B-cell clonal lines for further study. Here, a cluster is a set of clonally related BCR sequences, as defined by the standard or BRILIA annotation method. We compared the cluster sizes and counts returned by the standard and BRILIA methods. In Figures 5A,B, we compared how many clusters of one method were associated with the cluster of the other method, where an associated cluster shares at least one BCR sequence. We found that typically, a single BRILIA cluster is associated with a given standard cluster (Figure 5A), but that the converse is not true—multiple standard clusters are often associated with a given BRILIA cluster. These findings suggest that many standard clusters are a subset of BRILIA clusters.

In Figures 5C,D, we compare the differences in cluster sizes from one method’s cluster in relation to the other method’s largest associated cluster. We found that BRILIA clusters are larger than their associated standard clusters (Figure 5C), while standard clusters are generally smaller than their associated BRILIA clusters (Figure 5D). In summary, BRILIA clusters are systematically larger than their standard cluster counterparts, and thus, we expect to see more complex lineage trees based on BRILIA annotations.

### BRILIA Preserves Diverse Lineage Trees with High CDR3 Mutations

**Figure 6:**
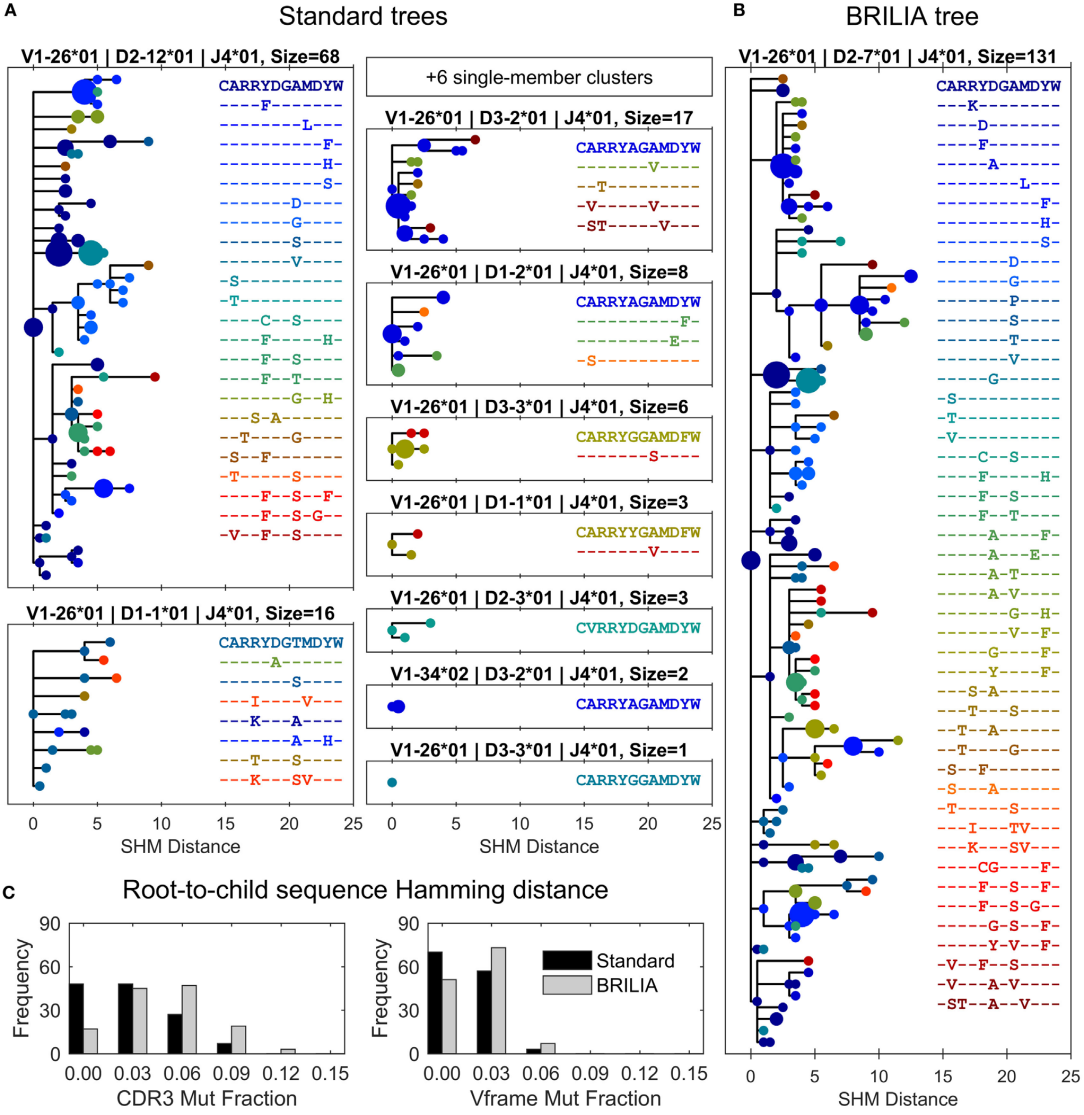
**Differences in lineage trees and somatic hypermutation (SHM) frequencies between the associated standard and BRILIA clusters from the example in Figure 5**. **(A)** Lineage trees are assembled from standard clusters that are subsets of a larger associated BRILIA cluster. The *x*-axis shows the absolute SHM distance, where the difference in SHM values between parent and child sequence is the SHM distance between the two sequences. Each dot color corresponds to a unique CDR3 sequence, and the dot size is scaled proportional to the sequence template count relative to the total template count within each lineage tree. The SHM distance is calculated based on the comparison of two 125-nucleotide sequences. Note that six single-member clusters are not drawn. **(B)** Lineage tree of a large BRILIA cluster that encompasses standard clusters. **(C)** Mutation frequencies of the V gene framework and CDR3 predicted by the two methods.

Different clustering results can ultimately translate to different lineage trees and interpretations of how affinity maturation has progressed within a clonal group. As an example, we compared lineage trees from the case where a large BRILIA cluster was represented by 15 separate standard clusters (red circles in Figure 5). Trees were drawn so that each unique BCR sequence was a circle, whose size reflected the sequence template count and whose color corresponded to a unique CDR3 sequence (Figure 6). We would expect trees in which clones with high template counts coincide with branch points because highly proliferating B cells are more likely to undergo SHM, generating diverse lineages. The largest tree given by the standard method shows general features of such a tree (Figure 6A), although the second standard tree (of size = 16) displays an unlikely scenario in which sequences are interlinked without an expanded B-cell clone and with a high CDR3 variability within the small cluster.

The corresponding BRILIA tree had leaves ending with low template–count clones that usually stemmed from larger clones (Figure 6B), and the tree was deeper and wider than the trees returned by the standard method. For the all BCR sequences in this example, BRILIA predicted a higher number of accumulated mutations in the CDR3 than when using the standard method (Figure 6C). This representative example illustrates how combining lineage tree assembly and gene annotation can result in substantially larger, richer B-cell lineage trees that are biologically plausible. It also shows how standard annotation methods can systematically underestimate the extent of SHM. While such clonal families make up a small percentage of the overall B cell repertoire, they may play a disproportionately important role in antibody responses to infection because they represent the most affinity-matured members of the repertoire.

### Insights into SHM Mechanisms

Proper mutation annotations can help to validate proposed mechanisms of SHM. Currently, the C ➔ T and G ➔ A mutation rates can be explained by AID-mediated deamination of C that creates C ➔ U mutations, which triggers MSH2/6, polymerase η, and uracil DNA glycosylase to fix U:G mismatches [recently reviewed by Casellas et al. (88)]. AID recognizes certain 3- or 4-nt long sequences called hot spots (51, 53, 81, 82); hence, one could identify SHMs caused by AID if the mutations occur at the signature hot spots. However, the A and T mutations occur at different 2-nt long hot spots (80), suggesting an alternate mechanism of mutation. There are two hotly debated theories of the mechanism underlying A and T mutations (89). One theory proposes that adenosine deaminase that acts on RNA (ADAR) converts adenosine to inosine, which occurs at a different hot spot and introduces A mutations mostly on the coding DNA strand (50, 67, 89). The other theory assumes that the AID-induced U:G mismatch triggers multiple DNA repair enzymes to eventually introduce A:T mutations nearby (51, 90, 91). Past studies identified SHM by comparing the V segment to the predicted germline V gene (92, 93); in contrast, here, we show SHM across the entire VDJ junction and based on inferred parent–child sequence relationships that better reflect the true nucleotide substitution frequencies. We present three findings on the mechanisms underlying SHM based on our analysis of the mice BCR sequencing data.

#### Molecular Mechanisms for A Mutations

**Figure 7:**
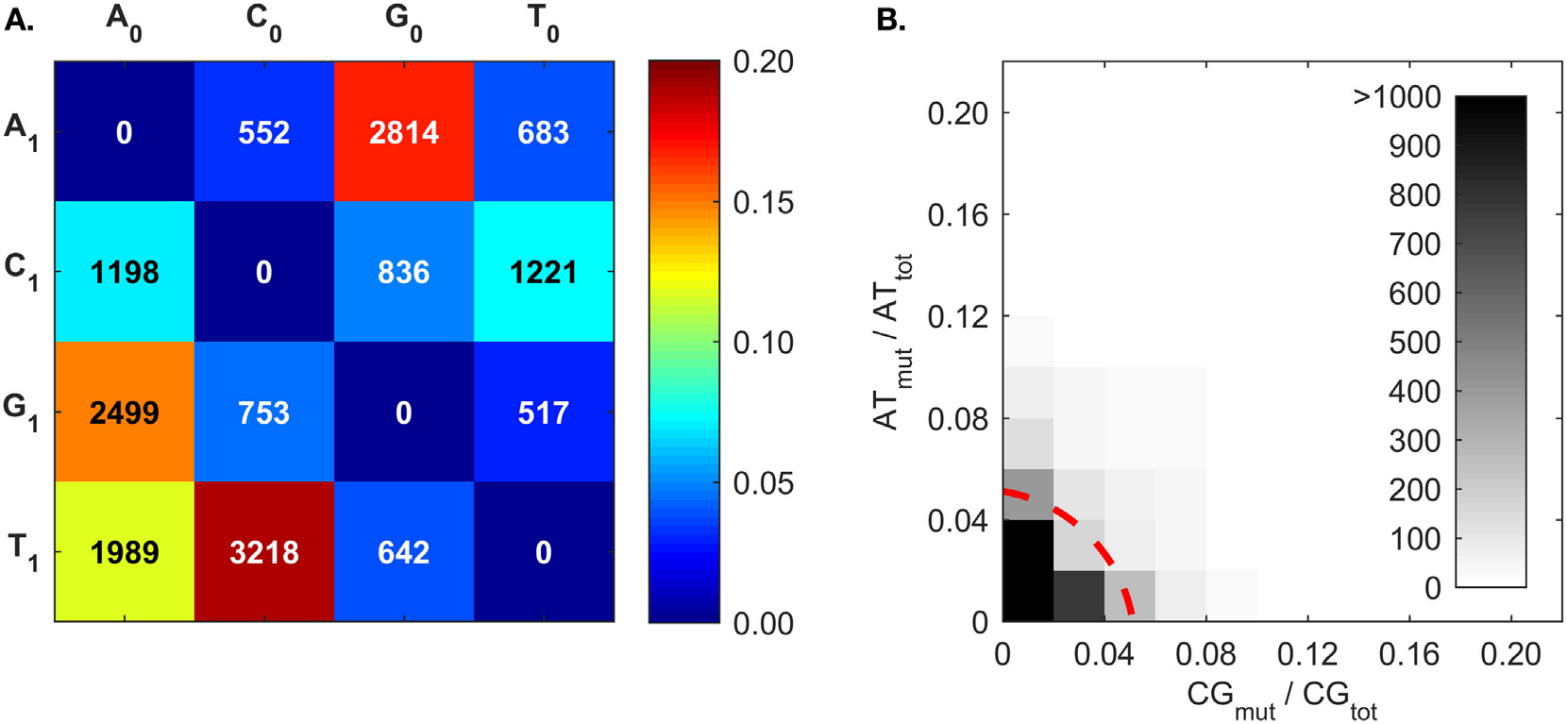
**Somatic hypermutation (SHM) frequencies returned by BRILIA, for the purpose of evaluating SHM mechanistic models**. **(A)** Cumulative frequency of SHM propensities for VDJ segments, excluding N regions. X_0_ is the parent nt and X_1_ is the child nucleotide. **(B)** The [A ➔ G + T ➔ C] mutation frequency (*AT_mut_*) normalized by the total A + T content (*AT_tot_*), plotted against the [C ➔ T + G ➔ A] mutation frequency (*CG_mut_*) normalized by the total C + G content in the VDJ segments (*CG_tot_*). The dotted red line, which depicts a circle with its center at the origin and a radius of 0.06, marks the mutation rate that captures 90% of the mutated sequences.

The mutation frequencies (Figure 7A) confirm the frequent C ➔ T and G ➔ A mutations generated by AID (50–53, 83), and also the higher A mutations over T mutations that reflect what is known as strand-biased mutations (67, 68, 94). The mutation frequencies of A follow the trend of G > T > C. To our best knowledge, this A mutation trend has not been previously discussed, and only the A ➔ G mutation was proposed to result from an inosine (I) intermediate during ADAR-mediated mutations [i.e., A ➔ I ➔ G (80)]. Interestingly, the A mutation trend coincides with that of I:X base-pairing free energy measurements (95). The Gibbs free energies of I:C, I:A, and I:G base pairs within a short dsDNA segment are −8.8, −7.5, and −6.3 kcal/mol, respectively (95); that is, inosine most closely resembles G, then T, and finally C. The explanation for frequent A mutations over T mutations is still being sought; the transcription of the BCR gene may provide an opportunity for A mutations in one DNA strand (67, 80, 94, 96, 97). There is a competing hypothesis that A:T mutations are the result of an AID-triggered patchwork repair process around C ➔ U mutation sites (51, 90, 91). In this case, given that C:G mutations would induce A:T mutations, we would expect a correlation between C:G mutations (*CG_mut_*) and A:T mutations (*AT_mut_*); this does not appear to be the case (Figure 7B).

#### Hot Spot Motifs

**Figure 8:**
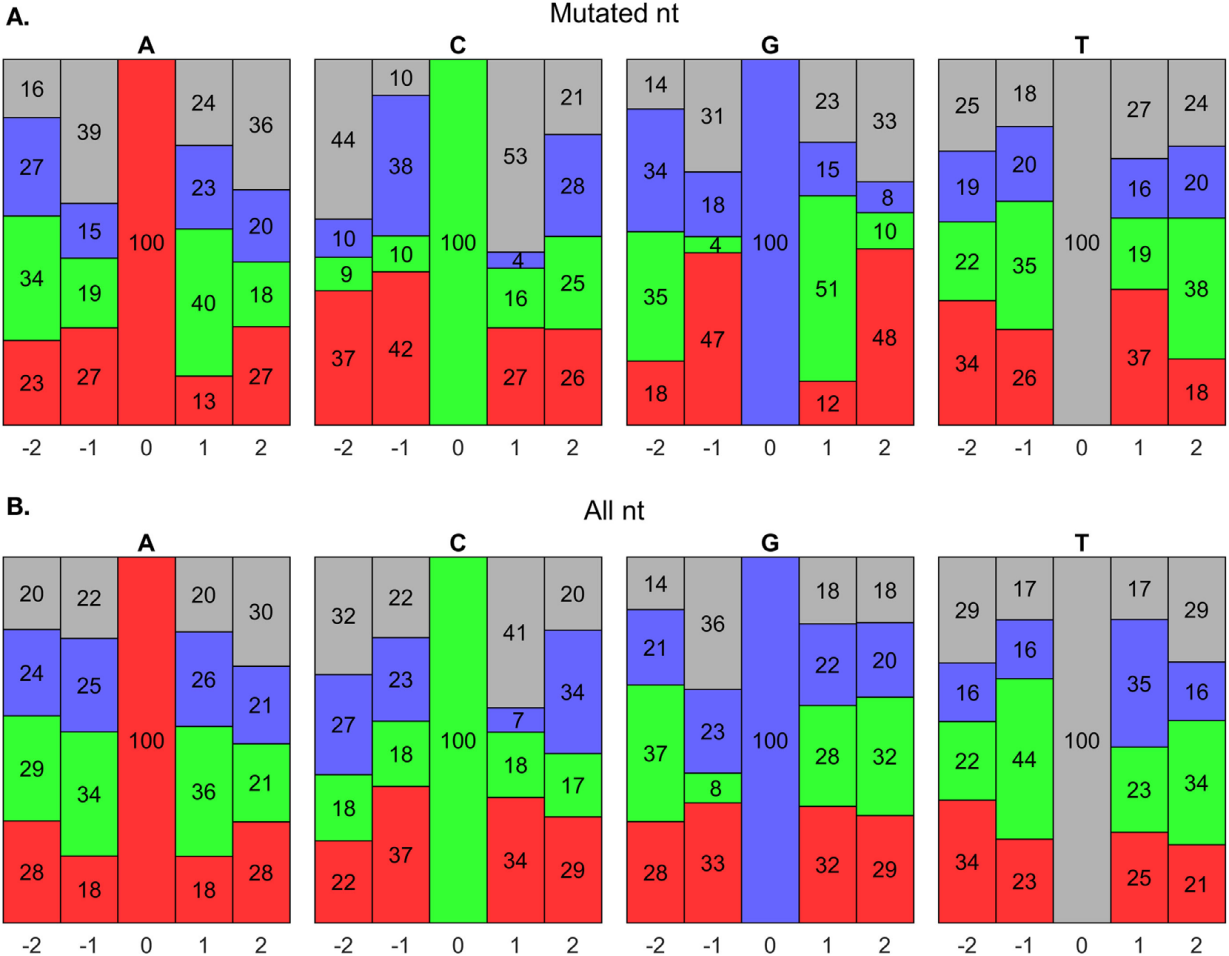
**Somatic hypermutation (SHM) hot spot analysis using BRILIA annotations**. **(A)** Evaluation of nucleotide (nt) compositions near only mutated nts, which are at the 0 positions. The negative and positive positions are nts toward the 5′ and 3′ sides, respectively, of the 0 position nt. The nt color codes are A = red, C = green, G = blue, and T = gray. **(B)** Evaluation of nt compositions of all nts, regardless of whether they mutated.

AID has been shown to mutate Cs near a WRCY (82), WGCW (51), WRCH (81), or WRC (53) hot spot motif, where W = A/T, R = A/G, Y = C/T, or H = A/C/T. We evaluated the composition of nts around mutated Cs in our data set and found that it initially agreed with a WGCW hot spot for AID (Figure 8A); however, we found that for any C, regardless of mutation, the + 1 position consistently contained Ws (Figure 8B). Hence, the WGCW (and potentially WRCY and WRCH) hot spots predicted by others may be simply arise from the fact that the +1 nt is biased toward a certain nt depending on the host species (53). Overall, we found that C mutations prefer the WGC motif, which is a subset of the WRC hot spot predicted by *in vitro* studies of AID mechanisms (53). The hot spot for G mutations is the complement sequence, GCW. Meanwhile, mutations of A have been previously shown to occur at WA hot spots (80). In support of this, our predicted hot spots for A and T mutations are TA and TA, respectively.

#### V Gene Mutations as a Proxy for CDR3 Mutations

**Figure 9:**
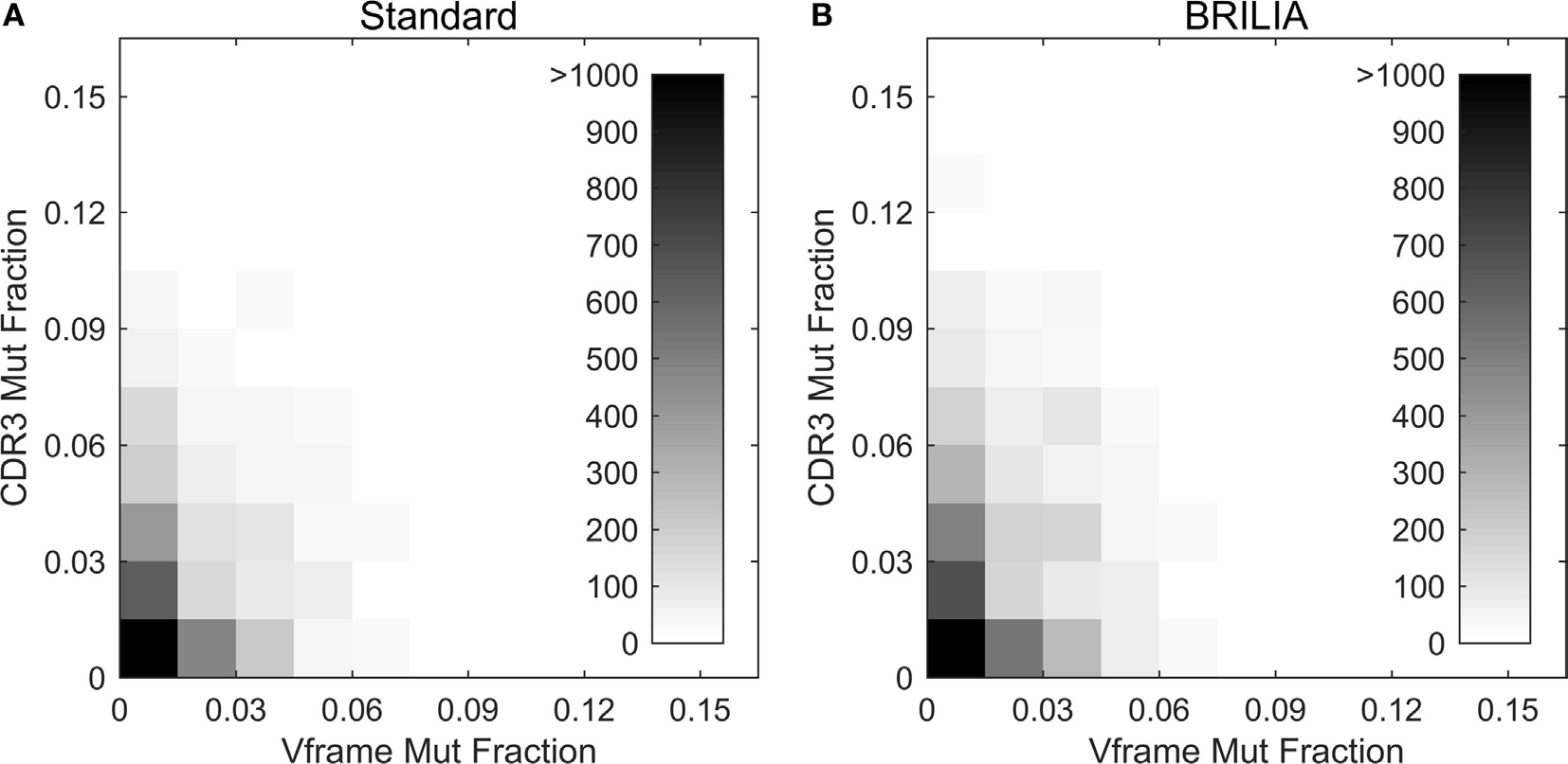
**Somatic hypermutations (SHM) in CDR3 and V framework regions**. Comparison of the mutations accumulated in the CDR3 versus V framework (Vframe) regions, as determined by **(A)** the standard method and **(B)** BRILIA.

Past studies often used the V gene mutation rates as a proxy for the CDR3 mutation rates (4, 5, 98) because resolving the germline D genes was difficult, especially when repertoire-wide sequencing data were unavailable. We investigated the correlation between mutations in the V gene framework (Vframe) and CDR3. Although SHMs are likely unfavorable in the Vframe region since it encodes conserved structural areas of the BCR (99), we still expected some level of silent mutations to correlate with SHMs in the CDR3. Results from BRILIA and the standard annotation methods show that there is a lack of correlation between SHM rates in the Vframe and CDR3 (Figure 9), suggesting that Vframe SHMs are a poor proxy for CDR3 SHMs. Given that the CDR3 region has the highest sequence diversity and typically accommodates the most SHM (14, 99), these findings demonstrate that it is critical to measure SHM frequency across the entire CDR3 to accurately assess the overall degree of SHM and affinity maturation in B cells.

## Discussion

We presented a novel approach to annotating and analyzing BCR sequencing data that leveraged repertoire-wide B-cell lineage information. By using simulated BCR sequencing data, BRILIA performed substantially better than existing method in annotating the D gene and identifying SHMs. We also showed that the identified SHMs from existing algorithms often provided biologically implausible results, such as inconsistent nt substitution frequencies between the V and the DJ segments. Finally, we applied BRILIA to real-life BCR sequencing data from splenic germinal center B cells of C57BL/6 mice. BRILIA yielded larger, more complex B-cell lineage trees compared to other methods.

Unlike common methods of determining SHM by comparing germline-child sequence pairs, BRILIA calculates SHM frequencies based on inferred parent–child relationships across the entire repertoire. The resulting SHM identification provided a more accurate description of SHM patterns, which was used to evaluate hypotheses related to SHM mechanisms, SHM hot spots, and extent of affinity maturation. Our results showed that there was a distinct order to A mutations (G > T > C), which supports the theory of an ADAR-based mutation mechanism *via* an inosine intermediate (80). Furthermore, we found that the hot spot motif associated with C mutations could most simply be described as WGC, which agrees with *in vitro* experiments on AID (53) and suggests that other more complex hot spots might be the result of intrinsic nucleotide position biases irrespective of mutations. Finally, we showed that SHM frequency in the V gene, a common proxy for overall SHM frequency in many B cell repertoire studies, was a poor predictor of SHM frequency in the highly variable CDR3. These findings highlight the importance of using repertoire-based, full VDJ annotations to evaluate the extent of affinity maturation of B-cell repertoires.

### BRILIA Helps Separate Real BCR Genes from Those Created by Sequencing Error

A persistent issue with analyzing high-throughput sequencing data is separating real sequences from those created by sequencing error. The ImmuniTree (46) and IMSEQ (36) algorithms address this issue, but completely removing sequencing errors is not always feasible. We expect that BCR sequences generated by error will most likely have low template counts and be assigned as “leaves” in the lineage tree. If the goal is to identify real BCR genes, and especially those from clonally expanded B cells, then this can be achieved by looking for sequences with higher-than-background template counts and are designated as lineage tree “nodes.” BRILIA helps identify lineage tree nodes and clonally expanded B cells by outputting the number of descendants associated with each sequence.

### Limitations of BRILIA

The consolidation of lineage trees, clustering, and annotation into a single algorithm makes BRILIA a powerful tool for immunosequencing research. However, limitations also stem from this strength, in that the cluster-based annotation scheme can underperform if the sequences are clustered incorrectly or if the root sequence is not correctly identified. For instance, BRILIA is not fully immune to accidentally grouping clonally unrelated sequences into the same cluster if a path is available or if the cutoff distance is set too large. We are investigating strategies to automatically determine the cutoff point and allow for variable cutoffs among different clusters. In addition, there are certain VDJ recombination events that BRILIA does not account for, including double D insertions [which creates VDDJ junctions (65)] and lack of D usage (which creates VJ junctions).

If, after the annotation process, multiple VDJ annotations are suggested, then BRILIA removes only pseudogene suggestions. Additional calculations to remove or prioritize the remaining degenerate annotations are not performed as this may bias the repertoire-wide VDJ gene usage frequencies. Processing longer sequences can help to reduce the occurrence of degenerate solutions.

### BRILIA Processing Speed

BRILIA can process large volumes of BCR sequences within a reasonable amount of time, even while determining lineage relationships among B cells. By using a 3.4 GHz quad-core processor with 16 GB of memory, BRILIA required 400 s to process our repertoire data with 12,300 sequences (or 31 ms per each 125-bp sequence). The overall computation time can be further reduced by splitting annotation jobs across more processors.

### BRILIA Input and Output Files

Although we focused on short 125-bp sequences in this study, BRILIA can process longer sequences that extend the full length of the V and J segments, including the CDR1, CDR2, FR1, FR2, and constant regions. If sequences contain the constant region attached to the J segment, BRILIA will trim the constant region. The input files for BRILIA are currently fasta, fastq, csv, xls, and xlsx files containing the sample name and sequence data. To supply the template count data for plotting lineage trees (as shown in Figure 6), tabulated data formats are preferred. Datasheets S1–S3 Supplementary Material show both example input and output files. BRILIA assumes that the input files contain contiguous sequences and not raw pair-end sequence reads. We recommend performing basic sequence formatting before running BRILIA to ensure most sequences are in the positive sense direction and span the VDJ junction.

### Concluding Remarks

In conclusion, we have demonstrated the ability of BRILIA to predict consistent SHM rates across the VDJ segments and its ability to identify clonally related sequences. These capabilities have wide utility for research related to tracking B-cell affinity maturation in a range of areas of research from infection and vaccination to autoimmune disorders and cancer. BRILIA is a powerful resource for processing and analyzing BCR sequences, and we are currently developing a publicly accessible web-based server for it. The BRILIA source code can be found in GitHub at https://github.com/BHSAI/BRILIA, and the stand-alone executable version is available on request. Please contact the corresponding author for technical support or further information.

## Author Contributions

DL, SC, and IK conceived the project idea, interpreted the results, and wrote the manuscript. SC, CC, SB, and AW acquired project funding. SC and AW oversaw the project. CC performed mouse experiments and reviewed the manuscript. DL developed BRILIA and processed the sequencing data.

## Conflict of Interest Statement

The authors declare that the research was conducted in the absence of any commercial or financial relationships that could be construed as a potential conflict of interest.

## Abbreviations

ADAR, adenosine deaminase acting on RNA; AID, activation-induced deaminase; bp, base pair; BCR, B-cell receptor; BRILIA, B-cell repertoire inductive lineage and immunosequence annotator; CDR, complementarity-determining region; HMM, hidden Markov model; IMGT, ImMunoGeneTics; nt(s), nucleotide(s); SHM, somatic hypermutation; TDT, terminal deoxynucleotidyl transferase.
