# Supplementary material for: BRILIA: Integrated Tool for High-Throughput Annotation and Lineage Tree Assembly of B-Cell Repertoires
Source: Front Immunol. 2017 Jan 17;7:681. doi: 10.3389/fimmu.2016.00681 (PMC5239784; doi:10.3389/fimmu.2016.00681)
Supplement: Supplementary file 1 [file Presentation_1.PDF]

Supplementary Material

BRILIA: Integrated tool for high-throughput annotation and lineage-tree assembly of B-cell repertoires

Donald W. Lee[1], Ilja Khavrutskii[1], Anders Wallqvist[1], Sina Bavari[2], Christopher L. Cooper[2], and Sidhartha Chaudhury[1*]

*** Correspondence:** Sidhartha Chaudhury: sidhartha.chaudhury.civ@mail.mil

[1]Biotechnology HPC Software Applications Institute (BHSAI), Telemedicine and Advanced Technology Research Center, U.S. Army Medical Research and Materiel Command, Fort Detrick, MD, USA

[2]Molecular and Translational Sciences, U.S. Army Medical Research Institute of Infectious Diseases, Frederick, MD, USA

Obtaining simulation parameters for BCR repertoires

To simulate a repertoire of B-cell receptor (BCR) genes, we required parameters that define the average number of nucleotides (nts) deleted from the VDJ genes, average N region lengths, N region nt compositions, and somatic hypermutation (SHM) propensities. We obtained these parameters for the C57BL/6 mice by using BRILIA to process the BCR sequence data provided by Collins et al. [1] (Accession Number PRJEB8745 from www.ebi.ac.uk/ena). The nt compositions of terminal deoxynucleotidyl transferase (TDT) are shown in **Figure S1A** for the N_{VD} and N_{DJ} regions, but after "flipping" the N regions to their complement sequence if there were more pyrimidines (CT) than purines (AG) because TDT adds mostly purines to one of the two DNA strands [2, 3]. **Figure S1B** shows the probability that nt X_0 mutates to nt X_1, which refer to as the SHM propensities. The list of simulation parameters are provided in **Table S1**. When simulating the human BCR genes, we adjusted some parameters according to the VDJ gene deletion averages and N region lengths determined by Souto et al. [4].

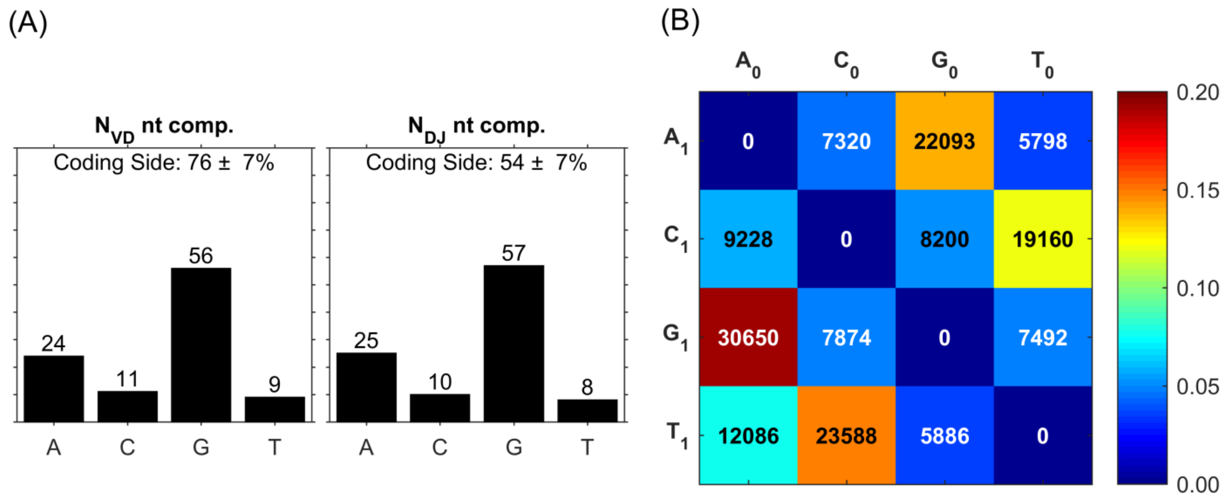

Figure S1 Nucleotide (nt) insertion by terminal deoxynucleotidyl transferase (TDT), as well as biases in somatic hypermutation (SHM) as obtained from the data set of Collins et al. [1]. (A) Composition of nts found in N regions, after taking the complements of an N region if the content of pyrimidines (CT) was higher than that of purines (AG). The frequency in which TDT is predicted to add to the coding DNA strand is provided in the top of the bar charts. (B) SHM propensities, or frequencies for observing nt X_0 mutate into nt X_1.

Table S1. List of parameters for BCR repertoire simulation.

<table>
<tr><th colspan="2">Parameter Name and Values</th><th>Mouse [1]*</th><th>Human [4]</th></tr>
<tr><td rowspan="4">VDJ Deletion</td><td>V3' AvgDel</td><td>1.0</td><td>2.1</td></tr>
<tr><td>D5'AvgDel</td><td>4.5</td><td>4.5</td></tr>
<tr><td>D3' AvgDel</td><td>3.4</td><td>5.0</td></tr>
<tr><td>J5' AvgDel</td><td>3.9</td><td>6.8</td></tr>
<tr><td rowspan="8">TDT Insertion</td><td>$N_{VD,}$ AvgNlen</td><td>3.8</td><td>7.0</td></tr>
<tr><td>$N_{DJ,}$ AvgNlen</td><td>2.9</td><td>7.0</td></tr>
<tr><td>N_{VD}, CodeSide</td><td colspan="2">0.75</td></tr>
<tr><td>N_{DJ}, CodeSide</td><td colspan="2">0.55</td></tr>
<tr><td>P_A</td><td colspan="2">0.25</td></tr>
<tr><td>P_C</td><td colspan="2">0.08</td></tr>
<tr><td>P_G</td><td colspan="2">0.60</td></tr>
<tr><td>P_T</td><td colspan="2">0.07</td></tr>
<tr><td rowspan="12">SHM Propensity</td><td>$P_{A \rightarrow C}$</td><td colspan="2">0.06</td></tr>
<tr><td>$P_{A \rightarrow G}$</td><td colspan="2">0.19</td></tr>
<tr><td>$P_{A \rightarrow T}$</td><td colspan="2">0.08</td></tr>
<tr><td>$P_{C \rightarrow A}$</td><td colspan="2">0.05</td></tr>
<tr><td>$P_{C \rightarrow G}$</td><td colspan="2">0.05</td></tr>
<tr><td>$P_{C \rightarrow T}$</td><td colspan="2">0.15</td></tr>
<tr><td>$P_{G \rightarrow A}$</td><td colspan="2">0.14</td></tr>
<tr><td>$P_{G \rightarrow C}$</td><td colspan="2">0.05</td></tr>
<tr><td>$P_{G \rightarrow T}$</td><td colspan="2">0.04</td></tr>
<tr><td>$P_{T \rightarrow A}$</td><td colspan="2">0.04</td></tr>
<tr><td>$P_{T \rightarrow C}$</td><td colspan="2">0.12</td></tr>
<tr><td>$P_{T \rightarrow G}$</td><td colspan="2">0.05</td></tr>
</table>

Abbreviations are as follows: AvgDel = average nt deletion length, LengthN = length of N region, CodeSide = fraction of N regions that were produced by TDT on the coding DNA strand, P_X = fraction of nt X inserted by TDT, and $P_{X0 \rightarrow X1}$ = fraction of nt X_0 that were mutated to nt X_1 (normalized over all 12 pairwise mutations).
* Processed sequence data with BRILIA.

Simulating human and mice BCR repertoires

Details of the procedure for generating simulated BCR repertoires are provided below. All parameter values are shown in **Table S1**.

1) Human and mouse VDJ germline genes were downloaded from IMGT. For mouse genes, we retained only those found in C57BL/6 mice. For human genes, we used the same database as that used to test the *partis* algorithm [5] to ensure a fair comparison of algorithm performance.
2) V, D (excluding D inverse), and J genes were randomly selected using a uniform random number, R.
3) Nucleotide deletions for gene edges were determined according to the exponential distribution function, Del = –AvgDel × ln(R).
4) N-region lengths were generated by the equation N_{length} = –AvgNlen × ln(R).
5) The nt compositions of N regions were generated to reflect those of TDT, which shows a high propensity to add G and A [2, 3].
6) To mimic elongation of the non-coding strand by TDT, we took the complement of the N region. The complement is take if the random number R is > CodeSide.
7) The V, N_{VD}, D, N_{DJ}, and J segments were concatenated into a single sequence. Nonproductive junctions were discarded and steps 1-6 were repeated until a productive junction was made.
8) Full length VDJ junctions were then trimmed and aligned so that the 118 Trp (TGG) of the CDR3 region was at the end, and the total nt count was 125 per sequence. These short sequences resemble those generated by several high-throughput sequencing platforms.
9) Each germline VDJ sequence was subjected to 5 iterations of mutations so that 5 nts were replaced at non-repeating locations to ensure maximum divergence. The nts were mutated from nt X_0 to nt X_1 according to the SHM propensity probabilities, $P_{X0 \rightarrow X1}$. Mutations that created stop codons were discarded. The steps for selecting and mutating a nt are as follows:
    a. Determine ACGT content for those that are allowed to be mutated.
    b. Multiply each nt content by the total probability of mutating that nt. This is defined as the nt mutation propensity, Q_X. There are 4 Q values: Q_A, Q_C, Q_G, and Q_T.
    c. Use a random number to select 1 of the 4 nt bases to mutate, based on the distribution of Q values. This initial nt is denoted as X_0.
    d. Use a random number to select the final nt, based on the distribution of $P_{X0 \rightarrow X1}$ values for the X_0 nt. Note that there are only 3 $P_{X0 \rightarrow X1}$ values per X_0 nt.
    e. Use a random number to select the position of X_0 to mutate amongst all available mutation locations.
    f. Accept the mutation if it generates no stop codon, or otherwise discard it.
10) Repeat all prior steps until 1000 germline sequences were generated, each having 5 mutated descendant sequences. The 1000 “germline” sequences and the 6000 “clonally expanded” sequences were saved in two separate files.

Resolving biases of TDT nucleotide insertion to improve VDJ annotations

The N-region refinement step in BRILIA exploits the “constraints” observed in the N regions, mediated by the preferential insertion of A and G to a single DNA strand [2, 3]. Other algorithms also take into account the N-region nt composition to improve their annotation results [6, 7], using the probability function provided by Basu et al [8], but they may not consider which of the two DNA strands was elongated by TDT. Detailed TDT insertion bias statistics could be beneficial for improving annotation algorithms and for re-evaluating the level of diversity expected from VDJ recombination events. Therefore, we analyzed both the TDT strand direction and nt composition biases for our mouse data set.

Considering only the N-region composition of the coding strand (**Figure S2A**), we confirmed the bias toward G and C counts, as reported previously [2, 3]. Dinucleotide probabilities were also higher for GG and CC pairs than those expected from multiplying individual probabilities, suggesting that TDT elongates homopolymers [3]. However, if we assume that TDT inserts mainly purines (A and G) into either the coding or noncoding strand, we can assess “TDT nt insertion compositions” as opposed to “N-region nt compositions.” For this, we took the complements of N regions if the pyrimidine content was greater than the purine content, prior to extraction the nt compositions; we will refer to this procedure as “flipping” the N region. If both purine and pyrmidine contents were equal, we counted the strand elongation direction as “uncertain,” and these uncertain directions became the error values for flipping rates.

Overall, we found very different nt compositions after flipping the N regions (**Figures S2B,C, and D**), and the dinucleotide probability matrix was essentially the product of the frequencies of the two nts. As an example, for the N_{VD} region, P_{GG} = 0.39, which is close to $P_G^2 = 0.59^2$ = 035. Interestingly, 77% of the N_{VD} regions appear to stem from TDT adding to the coding strand, whereas the probability is much closer to evenly distributed for the N_{DJ} regions (55%). This bias in strand elongation direction for N_{VD} is interesting and could reflect either a preference for AG content in the N_{VD} region, or difference in TDT accessibility to the coding versus noncoding strand.

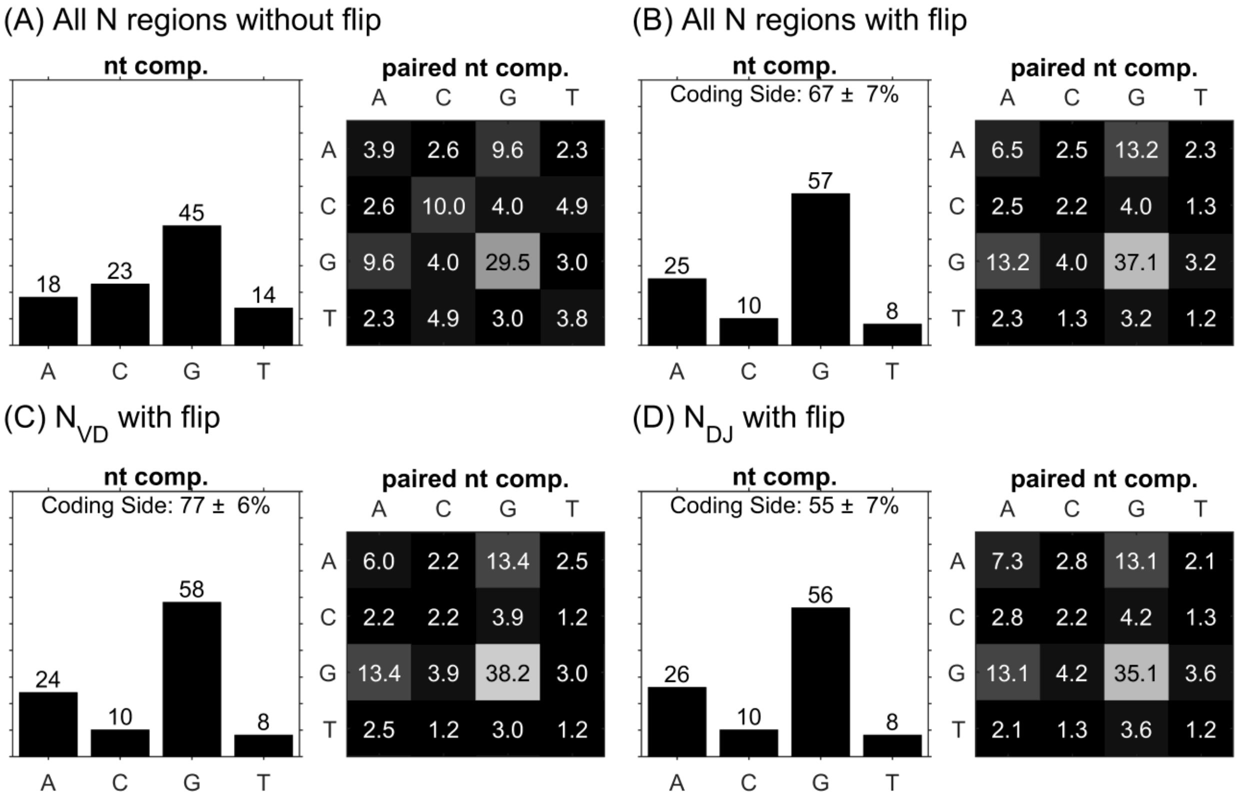

Figure S2 Composition of N region nucleotides and dinucleotides. (A) Compositions of the N_{DV} and N_{DJ} regions on the coding strand only. This shows the overall composition but does not reflect the bias of nt addition by TDT. (B) Similar to panel A data, but the N regions were "flipped" to their complement sequence if the CT content was higher than the AG content. The error in the N-region flip % is merely the % of N regions for which the CT content and AG content were equal. (C) Same data as panel B, but for the N_{VD} region. (D) Same data as panel B, but for the N_{DJ} region.

References

1. Collins, A.M., et al., *The mouse antibody heavy chain repertoire is germline-focused and highly variable between inbred strains.* Philosophical Transactions of the Royal Society of London B: Biological Sciences, 2015. **370**(1676).
2. Kepler, T.B., et al., *Interdependence of N nucleotide addition and recombination site choice in V(D)J rearrangement.* The Journal of Immunology, 1996. **157**(10): p. 4451-7.
3. Gauss, G.H. and M.R. Lieber, *Mechanistic constraints on diversity in human V (D) J recombination.* Molecular and Cellular Biology, 1996. **16**(1): p. 258-269.
4. Souto-Carneiro, M.M., et al., *Characterization of the Human Ig Heavy Chain Antigen Binding Complementarity Determining Region 3 Using a Newly Developed Software Algorithm, JOINSOLVER.* The Journal of Immunology, 2004. **172**(11): p. 6790-6802.
5. Ralph, D.K. and F.A. Matsen IV, *Consistency of VDJ rearrangement and substitution parameters enables accurate B cell receptor sequence annotation.* PLoS computational biology, 2016. **12**(1).

6. Munshaw, S. and T.B. Kepler, *SoDA2: a Hidden Markov Model approach for identification of immunoglobulin rearrangements.* Bioinformatics, 2010. **26**(7): p. 867-872.
7. Gaëta, B.A., et al., *iHMMune-align: hidden Markov model-based alignment and identification of germline genes in rearranged immunoglobulin gene sequences.* Bioinformatics, 2007. **23**(13): p. 1580-1587.
8. Basu, M., M.V. Hegde, and M.J. Modak, *Synthesis of compositionally unique DNA by terminal deoxynucleotidyl transferase.* Biochemical and biophysical research communications, 1983. **111**(3): p. 1105-1112.
